# Tacrolimus Dose Requirement in Iranian Kidney Transplant Recipients within the First Three Weeks after Transplantation

**Published:** 2016-08-01

**Authors:** S. Dashti-Khavidaki, S. Ghaffari, M. Gohari, M. R. Khatami, Z. Zahiri

**Affiliations:** 1 Nephrology Research Center, Tehran University of Medical Sciences, Tehran, Iran; 2 *Faculty of Pharmacy, Tehran University of Medical Sciences, Tehran, Iran*

**Keywords:** Iran, Kidney transplantation, Tacrolimus, Immunosuppressive Agents, Pharmacokinetics

## Abstract

**Background:**

Tacrolimus is the main immunosuppressive agent in many kidney transplant protocols with an initial recommended daily dose of 0.2 mg/kg of ideal body weight (IBW). However, due to the high inter- and intra-patient variability in its pharmacokinetics, the required tacrolimus doses may differ markedly from patient to patient.

**Objective:**

To assess the required tacrolimus dose to achieve the desired whole blood concentration within the first three weeks after kidney transplantation among Iranian patients.

**Methods:**

This cross-sectional study was performed at kidney transplantation ward of Imam Khomeini Hospital Complex where almost all patients receive thymoglobulin induction therapy and a calcineurin inhibitor, mainly tacrolimus, plus mycophenolate, and prednisolone as maintenance immnosuppressive drugs with the target tacrolimus whole blood concentration of 8–12 ng/mL for the first month after transplantation.

**Results:**

The mean±SD administered daily dose of tacrolimus during the first three weeks after transplantation was 0.085±0.024 mg/kg of IBW that resulted in a mean±SD whole blood concentration of 10.34±5.44 ng/mL. The required mean±SD dose of the drug to achieve the desired whole blood level of 8–10 ng/mL was 0.08±0.02 mg/kg. Only 27.4% of the assessed tacrolimus blood levels were within the desired range. Compared with males, females needed 19% more daily dose of tacrolimus to reach similar whole blood levels. Tacrolimus blood levels were significantly correlated with daily tacrolimus doses (r=0.307, p=0.001) and patients’ age (r=0.283, p=0.003).

**Conclusion:**

It seems that Iranian kidney transplant recipients need lower daily doses of tacrolimus to achieve the desired whole blood levels; compared with males, females need a higher dose.

## INTRODUCTION

Calcineurin inhibitors, cyclosporine and tacrolimus, are the cornerstones of immunosuppressive therapy in kidney transplant recipients, especially within the first three months after transplantation. Compared to cyclosporine-based immunosuppressive regimens, tacrolimus-containing regimens accompany less acute rejection episodes [1]. Due to the high inter- and intra-patient variability in pharmacokinetics of tacrolimus, the required tacrolimus maintenance doses may differ between transplant centers, transplantation type, and patient population [2]; however, based on average pharmacokinetic parameters, the initial recommended daily dose of tacrolimus in kidney transplant recipients is 0.2 mg/kg that have to be administered q12h [3]. To avoid high blood concentrations, it is recommended to adjust tacrolimus dose based on ideal body weight (IBW) in overweight patients [4]. Doses are subsequently adjusted to reach the desired blood trough concentrations. Tacrolimus have to be taken on a consistent manner of day meals and time [2]. Compared to adults, pediatric patients usually require higher tacrolimus doses to minimize variability in drug exposure due to higher drug clearance [2]. 

Variability in tacrolimus exposure is a known contributing factor to long-term graft loss [5]. Tacrolimus is a major substrate for intestinal and hepatic cytochrome P450-3A4 isoenzyme and P-glycoprotein. Therefore, its absorption and body exposure are mainly influenced by genetic factors and drug-food, drug-drug, and drug-herb interactions. All these variances make therapeutic drug monitoring necessary especially during the first 2–3 weeks after transplantation [2, 6]. Tacrolimus whole blood concentration has to be monitored 3–5 days after drug initiation, dose adjustment, or adding/discontinuing of known CYP450-3A4 inducers/inhibitors [7]. 

The objective of this study was to assess the required tacrolimus doses to achieve the desired whole blood concentration within the first three weeks after kidney transplantation among Iranian patients.

## PATIENTS AND METHODS

Setting

This cross-sectional study was conducted from September 2013 to December 2014 at Kidney Transplant Ward of Imam Khomeini Hospital Complex affiliated to Tehran University of Medical Sciences, Tehran, Iran. 

Immunosuppressive Regimen and Sampling

In our center almost all patients receive thymoglobulin induction therapy starting one hour before transplantation. Intravenous methyl-prednisolone is administered at doses of 500 mg, 250 mg, and 125 mg, respectively for day 1, 2, and 3 of transplantation; it is followed by 1 mg/kg/day oral prednisolone with rapid taper down to 5 mg/day after one month of kidney transplantation. A calcineurin inhibitor, mainly tacrolimus, plus mycophenolate and prednisolone are maintenance immunosuppressive drugs in this center. Tacrolimus is usually started with half needed daily dose during thymoglobulin induction and increased to a full dose 24 hours before suspected thymoglobulin termination time. All patients received Prograf^®^ (Astellas, Japan). In our center, the target tacrolimus whole blood concentration is 8–12 ng/mL within the first month after transplantation. All patients receive gancicloir/valganciclovir, cotrimoxazole, and clotrimazole as their routine prophylaxis against viral infections, *Pneumocystis jiroveci* pneumonia, and fungal infections. Patients are advised to receive their tacrolimus doses 2 hours after breakfast and dinner at 9:00 am and 9:00 pm. Consumption of foods and fruits that have considerable interactions with tacrolimus (grapefruit, high amount of bitter orange, and Earl Gray tea) are prohibited. Tacrolimus whole trough blood concentrations are assessed using monoclonal radioimmunoassay on samples taken before morning tacrolimus doses in a steady state.

The study protocol was approved by Local Ethics Committee of Tehran University of Medical Sciences. All patients signed informed consent forms. 

Data Analysis

Data were analyzed by SPSS^®^ for Windows^®^ ver 18. Normal distribution of data was assessed using one-sample Kolmogorov-Smirnov test. The required tacrolimus doses to achieve the desired whole blood concentration in patients were assessed using descriptive analysis and compared between males and females by *Student’s t* test for independent samples. Pearson’s or Spearman’s correlation coefficients were used to assess correlations between quantitative variables. A p value <0.05 was considered statistically significant.

## RESULTS

During 15-month study in our center, 54 newly kidney transplant recipients (24 females and 30 males) with a mean±SD age of 40.0±13.8 years received tacrolimus as their maintenance immunosuppressant and were included in this analysis. One-hundred and six whole blood concentrations were obtained during patients’ hospitalization after operation up to three weeks after transplantation, *i.e.*, almost two tacrolimus blood level per patient. The mean±SD administered tacrolimus daily dose during the first three weeks after transplantation was 0.085±0.024 (range 0.024–0.154) mg/kg of IBW that resulted in a mean±SD whole blood concentration of 10.34±5.44 (range 2–30) ng/mL. Compared with males, females needed 19% more daily doses of tacrolimus to reach similar whole blood levels (Table 1).

**Table 1:** Comparing females and males regarding tacrolimus dose and blood concentrations. Values are mean±SD

	All patients	Females	Males	p Value*
Considering all tacrolimus doses and blood levels				
Tacrolimus dose (mg/kg)	0.085±0.024	0.093±0.023	0.078±0.023	0.03
Tacrolimus trough level	10.34±5.44	10.37±5.06	10.31±5.78	0.753
Excluding tacrolimus doses and levels when patients receiving half tacrolimus doses				
Tacrolimus dose (mg/kg)	0.088±0.024	0.094±0.023	0.083±0.020	0.015
Tacrolimus trough level	10.69±5.44	10.50±5.03	10.87±5.83	0.742

* Comparing females and males

Considering whole blood tacrolimus concentrations of 8 to 12 ng/mL as the target blood level within the first month after kidney transplantation in this center, only 29 assessed tacrolimus blood levels (27.4% of tacrolimus blood levels) were within the desired range while 44 levels (41.5%) were lower and 33 (31.1%) were higher than the desired values. Daily tacrolimus doses that resulted in lower than the desired, and higher than the desired tacrolimus blood concentrations are shown in Table 2.

**Table 2:** Tacrolimus daily doses that resulted in different ranges of tacrolimus whole blood concentrations. Values are mean±SD (range).

Tacrolimus level (ng/mL)	Tacrolimus daily dose (mg/kg IBW)
<8	0.08±0.02 (0.03–0.13)
8–12	0.08±0.02 (0.05–0.15)
>12	0.10±0.02* (0.06–0.14)

* Significantly (p=0.003) different from the means of other groups.

During this survey, the only concomitant administered drug with reported drug interaction with tacrolimus was diltiazem that is an inhibitor for CYP 450-3A4/3A5 and P-glycoprotein [7, 8]. Eighteen (33%) out of these 54 patients were taking diltiazm. The mean±SD daily dose of tacrolimus did not significantly (p=0.604) differ between diltiazem users (0.083±0.024 mg/kg) and non-users (0.085±0.024 mg/kg). With the same mean daily tacrolimus dose, the mean±SD trough blood concentration of tacrolimus was non-significantly (p=0.085) higher among diltizaem users (11.58±5.23 ng/mL) than non-users (9.85±5.48 ng/mL). Although this difference in tacrolimus blood level did not reach statistical significance, it seems to be clinically important. 

Tacrolimus blood level was significantly correlated with the daily tacrolimus dose based on mg/kg of IBW (r=0.307, p=0.001) and patients’ age (r=0.283, p=0.003). Tacrolimus daily dose had a significant (p=0.037) negative correlation (r=0.203) with diltiazem daily dose.

## DISCUSSION

This study assessed the required maintenance dose of tacrolimus among Iranian kidney transplant recipients. The results showed that the mean tacrolimus daily dose to achieve the desired whole blood concentrations of 8–12 ng/mL within the first three weeks after kidney transplantation among Iranian patients was about 0.08 mg/kg of IBW that is lower than that recommended by the manufacturer and international references [3]. A recent report from a large kidney transplant ward in Australia also showed administering 0.15 mg/kg/day of tacrolimus for achieving blood levels of 7–8 ng/mL that is almost two times higher than the required doses in our patients [9]. Although it has been noted that over time, after transplantation, absorption of immunosuppressive drugs increases and necessary doses decrease [2], however, requiring low tacrolimus doses to attain the desired blood concentrations early after kidney transplantation in this study may be explained by inhibition of CYP 450-3A4 and P-glycoprotein by high methyl-prednisolone dose used in immunosuppression protocol in our center that may result in decreased pre-systemic tacrolimus metabolism and efflux [10]. Post-operative inflammation, anesthesia/opioid effects on intestinal motility, low diet intake during the first days of transplantation may also justify high tacrolimus concentration in some patients early after kidney transplantation [11]. Although some researchers noted no relationship between tacrolimus pharmacokinetics and patients’ sex [12], we found that females need higher daily doses of the drug by about 20% to achieve similar whole blood concentrations. A Serbian study also showed lower tacrolimus exposure in females compared with males after administering the same dose of 0.05 mg/kg of tacrolimus [13].

About one-third (31%) of our patients had higher than desired tacrolimus whole blood levels. These patients were administered significantly higher doses of tacrolimus compared with patients who achieved the desired or lower than the desired tacrolimus blood levels (Table 2). The main reason for achieving higher tacrolimus blood concentrations was increasing patients’ doses to about 0.1 mg/kg/d by nephrology fellows without considering previous patients’ tacrolimus blood levels. Since nephrotoxicity is a common side effect of calcineurin inhibitors including tacrolimus [3], determining the average required dose of tacrolimus in each kidney transplant population is of paramount importance to avoid toxicity.

This study suffers some limitations. The main limitation is the single-center nature of this study. Because different kidney transplant centers use different immunosuppression protocols that may interact with tacrolimus pharmacokinetics, data from other Iranian kidney transplant centers are required to confirm the proposed required daily dose of tacrolimus in these patients. Additionally, due to limited sample size, we were unable to compare different Iranian ethnicities (Tork, Kord, …) in terms of the required doses of tacrolimus. Larger studies are needed to distinguish possible differences between these ethnicities. 

It seems that to achieve the desired whole blood concentrations, Iranian kidney transplant recipients need a lower daily dose of tacrolimus compared to those recommended by manufacturer and international references. Compared with males, females need a higher tacrolimus dose. 

## Notes

**CONFILICTS OF INTEREST:** None declared.

## Notes

**FINANCIAL SUPPORT:** None.
